# Impact of Transgenic *Brassica napus* Harboring the Antifungal Synthetic Chitinase (NiC) Gene on Rhizosphere Microbial Diversity and Enzyme Activities

**DOI:** 10.3389/fpls.2017.01307

**Published:** 2017-07-25

**Authors:** Mohammad S. Khan, Syed U. Sadat, Asad Jan, Iqbal Munir

**Affiliations:** Genomics and Bioinformatics Laboratory, Institute of Biotechnology and Genetic Engineering (IBGE), Faculty of Crop Production Sciences, University of Agriculture Peshawar, Pakistan

**Keywords:** brassica, chitinase, rhizosphere microbes, environmental risk assessment, enzyme activities

## Abstract

Transgenic *Brassica napus* harboring the synthetic chitinase (*NiC*) gene exhibits broad-spectrum antifungal resistance. As the rhizosphere microorganisms play an important role in element cycling and nutrient transformation, therefore, biosafety assessment of *NiC* containing transgenic plants on soil ecosystem is a regulatory requirement. The current study is designed to evaluate the impact of *NiC* gene on the rhizosphere enzyme activities and microbial community structure. The transgenic lines with the synthetic chitinase gene (*NiC*) showed resistance to *Alternaria brassicicola*, a common disease causing fungal pathogen. The rhizosphere enzyme analysis showed no significant difference in the activities of fivesoil enzymes: alkalyine phosphomonoestarase, arylsulphatase, β-glucosidase, urease and sucrase between the transgenic and non-transgenic lines of *B. napus* varieties, Durr-e-NIFA (DN) and Abasyne-95 (AB-95). However, varietal differences were observed based on the analysis of molecular variance. Some individual enzymes were significantly different in the transgenic lines from those of non-transgenic but the results were not reproducible in the second trail and thus were considered as environmental effect. Genotypic diversity of soil microbes through 16S–23S rRNA intergenic spacer region amplification was conducted to evaluate the potential impact of the transgene. No significant diversity (4% for bacteria and 12% for fungal) between soil microbes of *NiC B. napus* and the non-transgenic lines was found. However, significant varietal differences were observed between DN and AB-95 with 79% for bacterial and 54% for fungal diversity. We conclude that the *NiC B. napus* lines may not affect the microbial enzyme activities and community structure of the rhizosphere soil. Varietal differences might be responsible for minor changes in the tested parameters.

## Introduction

Due to rapid increase in the world population, demand for edible oil is increasing day by day. *Brassica napus* (oilseed rape) is the third largest source of vegetable oil in the world (USDA, 2016). The productivity of oilseed rape is however, limited by several environmental factors including biotic stresses (Grover and Pental, 2003). Among biotic stresses, several fungal diseases are the major cause of low productivity of this crop. One way to overcome this problem is to develop fungal resistance in oilseed rape, through transformation. Transgenic *B. napus* plants were developed with the synthetic chitinase (*NiC*) gene that conferred resistance against fungal disease (Khan et al., 2013). In addition to the intended fungal resistance, impact assessment of the encoded proteins on rhizosphere microbial community structure and their enzymatic activities is an essential element of the environmental risk assessment of transgenic plants (Khan et al., 2016). Transgenic plants may presumably interact with rhizosphere microbes through root exudation and leaves or tiller degradation.

Before cultivation of transgenic plants for commercial purposes, the potential long-term environmental effects must be assessed through the procedures of environmental risk assessment (National Research Council, 2002). Environmental risk assessment under the guidelines of several regulatory authorities including the Cartagena Protocol on Biosafety is a regulatory requirement and is conducted on transgenic plants in a step-wise manner from laboratory to greenhouse, and then to confined and open field-testing (Watanabe et al., 2005; Baum and Madkour, 2006; Nickson, 2008).

Rhizosphere microbes such as fungi, and bacteria are essential part of the plant soil environment and carry out important biochemical reactions. Since, the soil microbes have a direct effect on plant growth and are important components of the local ecosystems (de Souza et al., 2015), impact of plant genotypes on rhizosphere microbial communities has been a vital element in many environmental risk assessment studies (Ikeda et al., 2006; Mimura et al., 2008). Several factors such as type of plant, soil condition, plant physiological state and changes in root exudates may influence soil microbial diversity and functions (Bossio et al., 1998; Griffiths et al., 1999; Yang and Crowley, 2000; Butler et al., 2003; Green et al., 2007). In addition, the transgene integration, expression and the related changes, both intended and unintended of transgenic plants may affect the rhizosphere microbes community structure and their functions. Previously, some studies reported alterations in the rhizospheric microbial properties and enzyme activities. For example negative effects were found on rhizospheric microbial activities caused by transgenic cotton plants (Chen et al., 2012). Transgenic maize with *Bt* gene had significant effects on the microbial community structure (Castaldini et al., 2005).

Many of the rhizosphere microbes contain chitin as an integral part of their cell walls. As a defensive strategy in transgenic plants, the use of genes encoding pathogenesis related (PR) proteins such as chitinases has been practiced for the last two decades. In addition to the antimicrobial activity against the target pathogen, there is an obvious possibility that the broad-spectrum antimicrobial proteins may also have direct or indirect impact on the rhizosphere microbial community structure and functions (Glandorf et al., 1995). Some studies were previously conducted to determine the effect of chitinases on soil microbiota in transgenic rice, Kiwi fruit and tobacco plants (Nakamura et al., 2004; Yuan et al., 2005; Wang et al., 2013). Transgenic rice and kiwi fruit plants were found with no significant effects on allelopathic and soil microbial structure and functions. However, the transgenic tobacco plants showed variable activities for some of the enzymes compared to the non-transgenic control plants (Wang et al., 2013). In addition, the constitutive production of chitinases in transgenic plants may also affect plant colonization by mycorrhiza such as vesicular-arbuscular (VA) mycorrhiza that contain up to 27% of chitin in their cell walls (Miller, 1993). This possibility has been tested in transgenic tobacco plants (Vierheilig et al., 1993; Tahiri-Alaoui et al., 1994). Irrespective of these findings, environmental risk assessment is based on a precautionary principle, practiced on case-by-case basis taking into consideration the nature of the plant, trait and the potential receiving environment.

Soil enzyme activity analysis has long been practiced to assess changes in microbial activities. Enzymes such as alkaline phosphomonoestarase, Arylsuphatase, β- glucosidase, Urease and Sucrase have been used for measuring the potential impact of transgenic plants on soil microorganisms (Lupwayi et al., 2007; Sun et al., 2007; Liu et al., 2008; Zeng et al., 2016). Changes in genetic diversity of microbial communities are often detected with molecular markers, specific for soil microbes. Molecular markers such as Ribosomal Intergenic Spacer Analysis (RISA) have been powerful tools to detect alterations in soil microbial diversity and community structure (Saito et al., 2008). These markers have also been used to compare transgenic plants with their non-transgenic control plants for differences in microbial community structure (Ikeda et al., 2006; Mimura et al., 2008; Sangeetha et al., 2015).

The present study is aimed to compare the transgenic *B. napus* (possessing the *NiC* gene) with non-transgenic lines for their potential impact on rhizosphere microbial communities and enzyme activities under greenhouse conditions.

## Materials and methods

The present work was conducted at Genomics and Bioinformatics Laboratory, Institute of Biotechnology and Genetic Engineering (IBGE), The University of Agriculture, Peshawar during 2015/2016.

### Plant material

Two locally developed *B. napus* varieties, Durre-NIFA (DN) and Abaseen-95 (AB-95) were previously transformed with the synthetic chitinase (*NiC*) gene at The Institute of Biotechnology and Genetic Engineering (IBGE), University of Agriculture Peshawar (Khan et al., 2013). The plasmid containing the synthetic chitinase (*NiC*) gene was kindly provided by Dr. Ikuo Nakamura, Graduate School of Horticulture, Chiba University, Japan. Six transgenic lines (DN-13, DN-120, DN-127, AB-11, AB-18, AB-31) and their parental non-transgenic varieties (DN and AB-95) were used for biosafety assessment in this study. Plants were grown in 5 kg pots containing soil-compost mix (50 w/w) in the greenhouse.

### Experimental design and soil sampling

Transgenic and non-transgenic lines of both DN and AB-95 varieties were grown in pots arranged in completely randomized design in the greenhouse. The experiment was conducted at the late vegetative stage of the plants and consisted of three replications with 5 pots of control and *NiC*-transgenic *B. napus* lines. *B. napus* seeds of T1 generation were sown in soil and the pots were regularly watered. Soil samples from the rhizosphere of the cultivated lines were collected at the late vegetative stage, sieved through a 2-mm sieve, homogenized and then stored at 4°C until further use. The soil samples were then used for microbial diversity and enzyme activity assays.

### Fungal analysis

#### Preparation of potato dextrose agar (PDA) media

Potato dextrose agar (PDA) media was prepared by boiling 250 g peeled and sliced potatoes in distilled water until the potatoes were soften. The broth was filtered by a cheese cloth. Required amount of distilled water was added to adjust the volume of the filtrate to 1 l, 10–20 g dextrose and 12–17 g agar were added to the medium. The medium was subsequently autoclaved for 15 min at 121°C and 15 PSI.

#### Preparation of fungal inoculums

The fungal infected leaves of *B. napus* were collected for inoculums preparation. The infected portion of leaves was separated properly. Three empty petri plates, two with distilled water and one with mercuric chloride were taken. The infected leaf portion of the leaf was clutched with the help of sterilized forceps and held in mercuric chloride for 30 s, then plunged twice in distilled water and placed carefully on the surface of PDA media, the petri plates were closed and labeled. The petri plates were incubated at 25°C for 7 days. Microscopic analysis of the fungal specimen on slides was conducted to confirm morphology of the fungus.

### Molecular analysis of the fungi

#### DNA extraction

The cetyltrimethylammonium bromide (CTAB) method was used for genomic DNA extraction from the fungal suspension culture as described by Knapp and Chandlee (1996).

### PCR amplification

The 18S rDNA of the fungal pathogen was amplified, using the general PCR with conserved primers as described by Jung et al. (2002). The 18S UP (5'-GACTGTGAAACTGCGAATGG-3'; AJ242597) and 18S DOWN (5′-TAAGTTTCAGCCTTGCGACC-3′; AJ242598), ITS UP (5′-GTCCTAACAAGGTTTCCGTA-3′) and ITS down (5′-TTCTCCGCTTATTGATATGC-3′) primers were designed for amplification of a rDNA fragment of about 1,000 size. PCR was carried out with a 50 ml reaction volume and the following solution were used in the master mix: template DNA, one nanogram (I ng), dNTPs (0.2 mM each dATP, dGTP, dCTP, and dTTP), primers (50 pcM), Tris-HCl (20 mM, pH 8.0), KCl (100 mM), EDTA (0.1 mM), DTT (1 mM), Tween 20 (0.5%), Non-idet P-40 (0.5%), glycerol (50%), 1 unit of *Taq* DNA polymerase (Takara, JAPAN), and MgCl2 (5 mM). PCR amplifications were carried out in a thermal cycler (i-cycler, Bio-Rad, USA). Denaturation of the genomic DNA was conducted at 94°C for 3 min. The PCR amplification was consisted of 40 cycles with denaturation for 40 s at 94°C, annealing for 60 s at 55°C, and extension for 60 s at 72°C for extension. Final extension was carried out at 72°C for 10 min.

### Protein assay

Leaves were collected from both transgenic and non-transgenic lines for protein extraction. About 500 mg of fresh leaf was taken from each individual plant and grinded with the help of mortar and pestle in liquid nitrogen until powder form was obtained. The sample was then transferred to eppendorf tube containing 500 μl protein extraction buffer. The sample was incubated at room temperature for 15 min with periodic vortexing. The mixture was then centrifuged at 25,000 × g for 15 min at 4°C to eliminate cell debris.

### Antifungal activity assay of extracted proteins

Proteins were extracted from leaf tissues for antifungal assay. A sample of 200 μl was taken from the supernatant and mixed with 2 μl of proteinase inhibitor. The fresh fungal suspension culture was then added to the extracted protein at a ratio of 1:9 and mixed thoroughly. The mixture was then spread on PDA plates. The plates were kept for incubation at room temperature in the growth chamber having a photoperiod of 16 h. Inhibition of fungal growth was then recorded in the control and transgenic plates. The experiment was conducted in two trials with three replications per trial.

### Quantitative real time-PCR analysis

Quantitative Real Time-PCR (qRT–PCR) was performed to determine the relative expression of *NiC* gene in *NiC* over-expressing transgenic and wild type control plants. Total RNA was isolated from leaves at early vegetative stage using RNeasy Plant Mini Kit (Thermoscientific, Germany). Around 5 μg total RNA was reverse transcribed into cDNA using superscript II (Invitrogen, USA). qRT–PCR analysis was carried out using ABI3700 system (Applied Biosystem, USA) as described by Nakashima et al. (2007). The *B. napus Actin* gene mRNA was used as an internal control. The *NIC* and *Actin* gene primers used were as follows:

Actin forward, 5′-TGAAGATCAAGGTGGTCGCA-3′, and reverse, 5′-AGAAGGCAGAAACACTTAGAAG-3′), *NIC* forward 5′-GGTCGATGCCGTCCTCCTGTCCTT-3′

*NIC* reverse 5′-CGCCTTGGTGGTGGTCTTGATGGT-3′.

### Rhizosphere enzyme activity assay

Five major soil enzymes i.e., alkaline phosphomonoestarase, Arylsuphatase, β-glucosidase, urease and sucrase were selected for assessment. Alkaline phosphomonoestarase: (EC 3.1.3.1) activity was determined according to Tabatabai and Bremner (1969). Fresh 1 g soil sample was collected from the rhizosphere of each line and was thoroughly mixed with 4 ml phosphate buffer (pH 8.0), 0.25 ml toluene and 1 ml of 0.115 M p-nitrophenyl phosphate (disodium salt hexahydrate) solution and was incubated for 1 h at 37°C. The formation of p-nitrophenol due to the activity of alkaline phosphomonoesterase was determined spectrophotometrically at 400 nm and the results were expressed as μg p-nitrophenol g^−1^ soil.

Arylsulphatase (EC 3.1.6.1) activity was measured according to Tabatabai and Bremner (1970). One gram freshly collected rhizosphere soil sample from roots of each selected line was incubated at 37°C for 1 h with 4 ml acetate buffer (pH 5.5), 0.25 ml toluene and 1 ml of 0.115 M p-nitrophenyl sulfate (potassium salt) solution. The formation of p-nitrophenol was determined spectrophotometrically at 400 nm and the results were expressed as μg p-nitrophenol g^−1^ soil.

β-Glucosidase (EC 3.2.1.21) activity was measured according to Eivazi and Tabatabai (1988). 0.25 ml toluene, 4 ml Tris (hydroximethyl) aminomethane buffer (pH 12) and 1 ml of 0.05 M p-nitrophenyl β-D-glucopyranoside solution were added to 1 g fresh rhizosphere soil sample, collected from each selected line. The samples were incubated for 1 h at 37°C. The formation of p-nitrophenol was determined spectrophotometrically at 400 nm and the results were expressed as μg p-nitrophenol g^−1^ sample.

For soil urease enzyme activity analysis, a 5 g soil sample was incubated with urea and then the enzyme activity was measured by determining ${\text{NH}}_{4}^{+}$-N using steam distillation (Tabatabai and Bremner, 1972). Sucrase activity was analyzed by measuring the soil glucose (C_6_H_12_O_6_) content as described (Tabatabai, 1994).

All determinations of enzymatic activity were performed in triplicate. Data collected from the analysis of soil enzymes were subjected to statistical analysis of variance using EXCEL/SPSS software.

### Rhizosphere microbial diversity assay

#### DNA extraction and ribosomal intergenic spacer analysis (RISA)

Rhizosphere microbial DNA was extracted from 0.5 g soil sample using the FastDNA SPIN Kit for soil (Qbiogene, Carlsbad, CA). Spacer region of ribosomal RNA was amplified from soil DNA using the specific primers ITSF/ITSReub for bacteria (Cardinale et al., 2004), and 2234C/3126T for fungi (Ranjard et al., 2001). These specific primers amplify the spacer region between the small (16S) and large (23S) subunits. Genotypic diversity was then determined based on sizes of the amplified fragments. The PCR mixture (20 μl) contained 2U of Taq DNA Polymerase (Takara, Japan), 1X PCR buffer (Takara), 0.5 μM each of forward and reverse primers, 200 μM of each dNTP, and 10 ng of template DNA. The PCR condition was as follow: 5 min at 94°C, 30 cycles of 1 min at 94°C, 1 min at 60°C, 2 min at 72°C, and a final extension for 7 min at 72°C. PCR products were run on 6% denaturing polyacrylamide gel with the GeneScan 2500 Red Dye (ROX) Size Standard (Applied Biosystems). The electrophoresis was run with 2,000 V for 2.5 h. Images of DNA fragments were visualized using molecular imager. The visualized bands were scored using Peak-scanner software (Bio-Rad Laboratories, Inc.). Statistical analysis was conducted for the number of bands appeared in each line and analysis of molecular variance (AMOVA) table was generated from the data collected. Along with statistical analysis, cluster analysis of the band data was also conducted using UPGMA method.

## Results

### Microscopic and molecular analysis of the causal fungi

**Figure 1:**
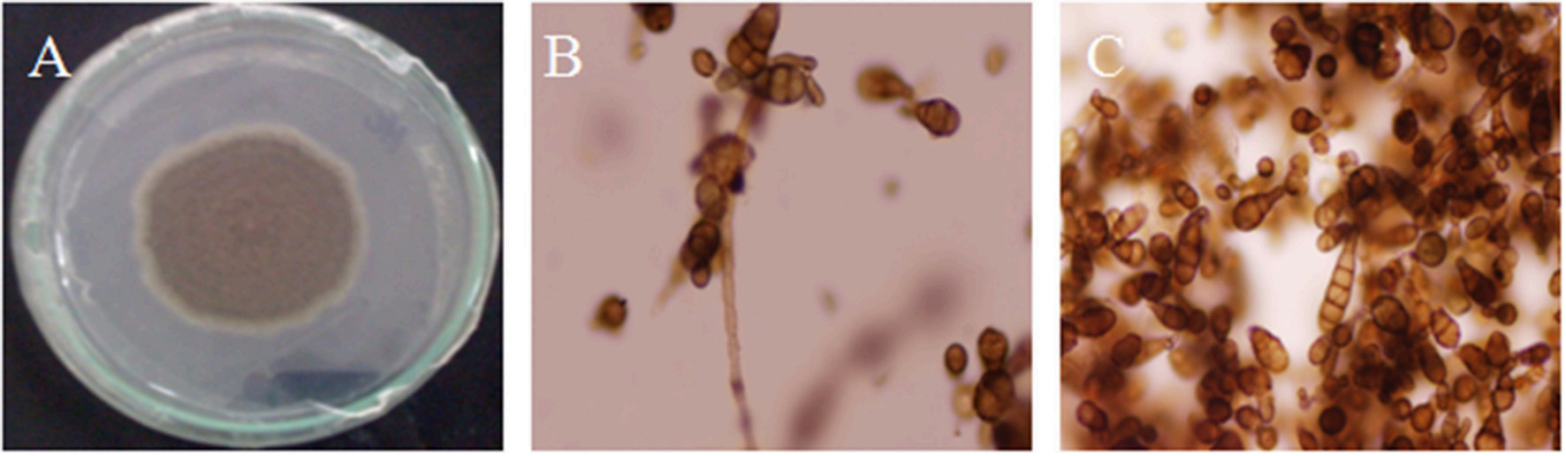
Morphological and microscopic observation of *Alternaria brassicicola*. Fungal growth on PDA plate **(A)**. Fungus showed brown hyphae ending in conidiophores **(B)** and individual conidia **(C)**.

The infected leaf samples were inoculated on PDA medium. Microscopic analysis was conducted to observe and identify the causal agent. The fungus produced abundant colonies with branched, septate and brownish mycelia. Conidiophores were simple, solitary or short in chain, olive-brown in color, septate and variable in length with terminal conidia. The mature conidia measured from 10–30 × 5–12 μm, short conical beakless. Based on the morphological characteristics of the conidia and mycelia (Figure 1), the causal agent was identified as *Alternaria brassicicola*.

**Figure 2:**
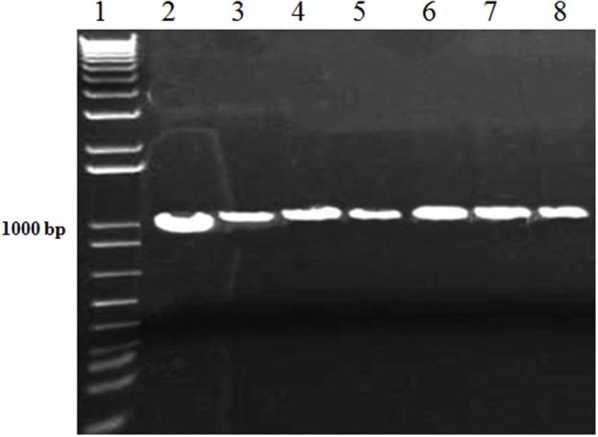
PCR of 18S rDNA of *A. brassicicola* with specific primers. Lane #1: 1Kb ladder; Lanes #2-8: Amplified products of 18S rDNA region from DNA samples of fungal innoculm.

**Figure 3:**
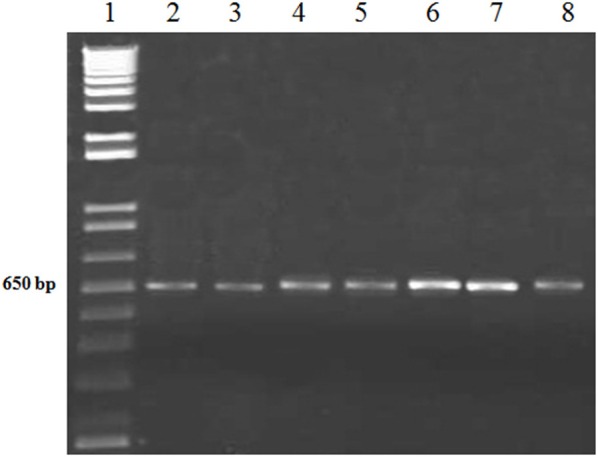
PCR of ITS region of *A. brassiscicola* with ITS specific primers. Lane #1: 1Kb ladder; Lanes #2-8: Amplified products of ITS region from DNA samples of fungal innoculum.

After the fungal pathogen detection with microscopic analysis, further confirmation of the causal fungi, *A. brassicicola* was carried out with the more reliable PCR method. The conserved sequences i.e., 18S and ITS rDNAs of the isolated fungi were amplified using specific primers for the 18S and ITS rDNAs. The amplified products were successfully resolved on agarose gel with estimated sizes of 1.0 and 0.6 kb for 18S and ITS, respectively, as shown (Figures 2, 3).

### Antifungal activity

**Figure 4:**
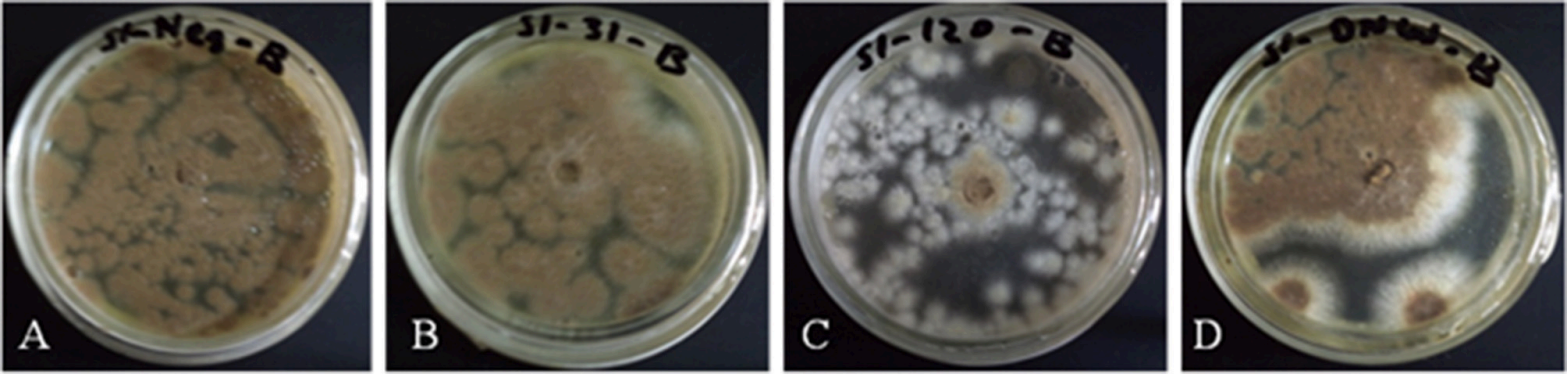
Antifungal activities of transgenic *B. napus* plants expressing the *NiC* gene. Fungal growth inhibition of *A. brassicicola* was observed on plates containing protein extracts from non-transgenic plants, DN **(A)**, AB-95 **(B)**, and transgenic lines, DN-120 **(C)**, AB-31 **(D)**.

Antifungal activity of crude proteins, extracted from leaves of transgenic and non-transgenic plants was evaluated against mycelial growth of *A. brassicicola*. The antifungal activities, expressed as (% growth inhibition) are shown (Table 1, Figure 4). The non-transgenic varieties, DN and AB-95 showed 0% inhibition. Transgenic lines of DN showed varied zones of inhibition among which DN-120 resulted in highest growth inhibition (30.9%), followed by DN-127 (7.79%) and DN-13 (2.85%) inhibition, respectively. Transgenic lines of AB-95 also showed different fungal growth inhibition. Line AB-31 showed highest growth inhibition (28.8%), followed by AB-18 (1.3%) and AB-11 (0.55%) respectively. These results revealed that some transgenic lines of both varieties have the potential of enhanced resistance against *A. brassicicola*.

**Table 1:** Measurement of fungal growth inhibition (%) by transgenic and non-transgenic lines.

**Varieties**	**Inhibition (%)**	**Varieties**	**Inhibition (%)**
DN	0	AB-95	0
DN-13	2.86	AB-11	0.56
DN-120	30.9	AB-18	1.3
DN-127	7.8	AB-31	28.8

### Expression analysis of the synthetic chitinase (*Nic*) gene

**Figure 5:**
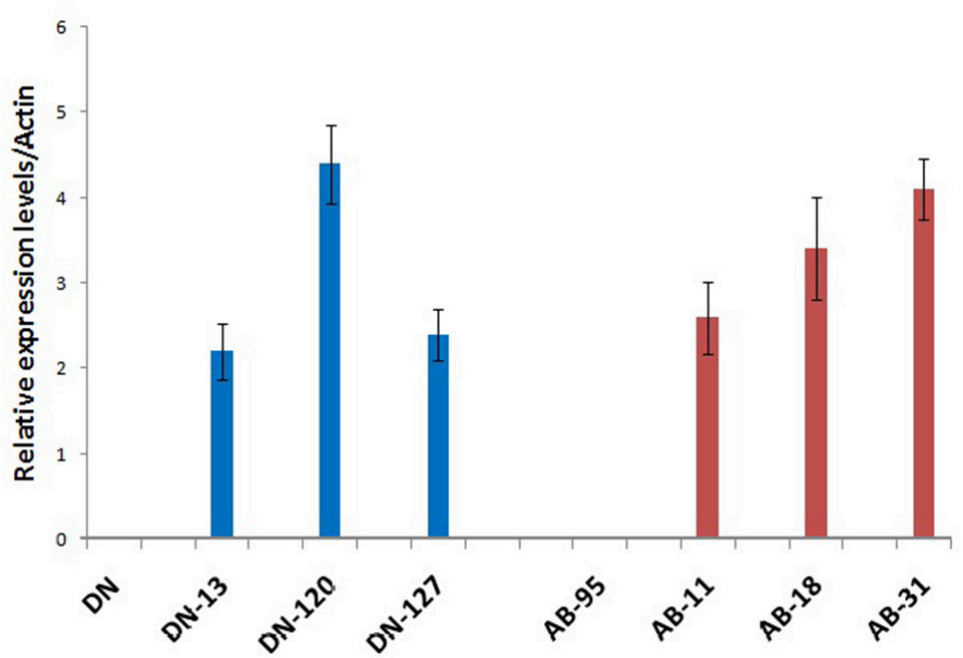
Quantitative RT–PCR Analysis of the *NiC* over-expressing transgenic and wild type control plants of DN and AB-95 *B. napus* varieties. Transgenic lines of both varieties showed expression of the *NiC* gene. No expression was observed in the wild type control plants. The *B. napus Actin* gene mRNA was used as internal control.

Real time quantitative PCR analysis of the *NiC* gene was conducted using all the transgenic and non-transgenic lines of the varieties DN and AB-95. Expression of the *NiC* gene was controlled by the Cauliflower Mosaic Virus (*CaMV35S*) constitutive promoter. All the transgenic lines of DN and AB-95 varieties showed expression of the *NiC* gene, while no expression was observed in the non-transgenic control lines (Figure 5). There was not much difference in the relative expression of the *NiC* gene in the transgenic lines of the two varieties. However, in comparison to other transgenic lines, the DN-120 and AB-31 showed high expression of the *NiC* gene. Comparatively higher expression of the *NiC* gene in these lines might correspond to their improved antifungal activities against the causal fungi, *A. brassicicola*.

### Effect of *Nic* transgenic *B. napus* on rhizosphere enzyme activities

The effects of *NiC* transgenic *B. napus* lines on the activities of five rhizosphere enzymes are shown (Tables 2, 3). The values of alkaline phosphomonoestarase activity in *NiC* transgenic lines of DN variety were from 434.48 ± 12.87 to 453.38 ± 1.67 μg g^−1^ dry soil, while the control line had value of 422.81 ± 4.58 μg g^−1^ dry soil. The alkaline phosphomonoestarase activity for the transgenic lines of AB-95 variety ranged from 436.98 ± 15.55 to 471.32 ± 5.00 μg g^−1^ dry soil, while the control line had value of 449.80 ± 8.22 μg g^−1^ dry soil. The arylsulphatase activity in *NiC* transgenic lines of DN variety were from 11.79 ± 1.40 to 16.75 ± 0.3 μg g^−1^ dry soil, while the control DN variety showed value of 12.59 ± 0.24 μg g^−1^ dry soil. The aylsulphatase activity in transgenic lines of AB-95 variety varied from 18.89 ± 0.59 to 41.45 ± 0.66 μg g^−1^ dry soil, while the control AB-95 showed value of 19.57 ± 0.18 μg g^−1^ dry soil. The β-glucosidase activity in the *NiC* transgenic lines of DN variety ranged from 128.23 ± 0.90 to 165.99 ± 1.12 μg g^−1^ dry soil, while the control DN showed a value of 135.74 ± 3.07 μg g^−1^ dry soil. For transgenic lines of AB-95 variety, the arylsulphatase activity varied from 358.62 ± 0.31 to 369.14 ± 0.41 μg g^−1^ dry soil, while the control AB-95 line had a value of 373.15 ± 0.11 ug g^−1^ dry soil. The urease activity in transgenic lines of DN variety ranged from 77.51 ± 4.32 to 85.86 ± 5.08 μg g^−1^ dry soil, while the control DN showed a value of 81.58 ± 3.36 μg g^−1^ dry soil. The transgenic lines of AB-95 had urease activities ranged from 80.69 ± 2.45 to 88.81 ± 4.14 μg g^−1^ dry soil, while the control AB-95 had a value of 85.62 ± 2.92 μg g^−1^ dry soil. The sucrase activity in transgenic DN lines varied from 0.23 ± 0.03 to 0.29 ± 0.02 μg C_6_H_12_O_6_· g^−1^ dry soil, while the control line showed a value of 0.23 ± 0.02 μg C_6_H_12_O_6_· g^−1^ dry soil, For transgenic AB-95 lines, the sucrase activity ranged from 0.24±0.03 to 0.30±0.04 μg C_6_H_12_O_6_· g^−1^ dry soil.

**Table 2:** Enzyme activities of Alkaline phosphomonoestarase (ALP), Arylsulphatase and β-glucosidase in soils of both transgenic and non-transgenic lines of DN and AB-95 varieties.

**Lines**	**Enzyme concentrations (ug g^−1^ soil)**				
	**ALP (μg g^−1^ soil)**	**Arylsulphatase (μg g^−1^ soil)**	**β-glucosidase (μg g^−1^ soil)**	**Urease (μg g^−1^ soil)**	**Sucrase (μg C_6_H_12_O_6_ g^−1^ soil)**
DN	422.81 ± 4.58	12.58 ± 0.24	135.74 ± 3.07	81.58 ± 3.36	0.23 ± 0.02
DN-13	447.78 ± 4.67	11.79 ± 1.40	167.06 ± 2.45	77.51 ± 4.32	0.26 ± 0.02
DN-120	434.48 ± 12.87	15.64 ± 0.48	128.23 ± 0.90	85.86 ± 5.08	0.29 ± 0.03
DN-127	453.38 ± 1.67	16.75 ± 0.30	165.99 ± 1.12	79.70 ± 6.44	0.23 ± 0.03
Overall for DN lines	433.66 ± 8.26	14.19 ± 0.60	149.25 ± 1.89	81.16 ± 4.8	0.25 ± 0.03
AB-95	449.80 ± 8.22	19.57 ± 0.18	373.15 ± 0.11	85.62 ± 2.92	0.30 ± 0.02
AB-11	436.98 ± 15.55	18.89 ± 0.59	369.14 ± 0.41	86.95 ± 3.12	0.24 ± 0.03
AB-18	469.78 ± 5.88	29.93 ± 0.65	358.62 ± 0.31	80.69 ± 2.45	0.30 ± 0.04
AB-31	471.32 ± 5.00	41.45 ± 0.66	366.12 ± 0.51	88.81 ± 4.14	0.24 ± 0.04
Overall for AB lines	456.96 ± 8.66	24.96 ± 0.52	366.75 ± 0.34	85.52 ± 3.16	0.27 ± 0.03

*± indicates standard deviation*.

**Table 3:** Probability (*p*-value) according to the pairwise student *t*-test of differences in soil enzyme activities between transgenic and non-transgenic lines of DN and AB-95 varieties.

**Genotype**	**Pairwise comparison**		**ALP**	**Arylsulphatase**	**β-glucosidase**	**Urease**	**Sucrase**
	**Factor 1**	**Factor 2**					
DN	DN	DN-13	0.98232	0.98232	0.775756	0.98123	0.97321
	DN	DN-120	0.982069	0.982069	0.707786	0.87541	0.74634
	DN	DN-127	0.992354	0.992354	0.973511	0.99781	0.99864
AB-95	AB-95	AB-11	0.985193	0.985193	0.944299	0.99872	0.74572
	AB-95	AB-18	0.819622	0.819622	0.849126	0.75644	0.99263
	AB-95	AB-31	0.981562	0.981562	0.967133	0.87891	0.71323
Overall	DN	AB-95	0.095659	0.05659	0.965552	0.91475	0.97453

*P ≤ 0.05*.

The overall data showed a no significant difference (*P* ≤ 0.05) among the mean activities of all the five enzymes between transgenic and non-transgenic lines (Tables 2, 3). From the data analysis, no significant (*P* ≤ 0.05) variation was found for the five enzyme activities between transgenic and non-transgenic lines of both *B. napus* varieties. However, significant varietal differences were recorded for arylsulphatase activities of DN and AB-95 varieties i.e., 14.19 ± 0.60 μg g^−1^ and 24.96 μg g^−1^ soil, respectively. Similar trend was observed for β-glucosidase activity. On overall basis, the mean value of β-glucosidase activity for DN variety was observed relatively lower (149.25 ± 1.89 μg g^−1^ dry soil) than that of AB-95 which showed value of 366.75 ± 0.34 μg g^−1^ dry soil.

### Rhizosphere microbial diversity analysis

From the bands pattern of fungal and bacterial profiles, Bi-variate data was generated. A total of 42 and 23 genotypes of fungi and bacteria were observed in the RISA analysis of soil microbes of both transgenic and non-transgenic lines of DN and AB-95 varieties (Table 4). Genotypes which appeared in only one of the replications were considered as specific genotypes. Although some line-specific genotypes were observed in both fungal and bacterial RISA but no specific genotypes were replicated thrice in replications of the assay. In DN variety, a total of 16 fungal and 8 bacterial genotypes were observed where the non-transgenic DN line was scored for 5 fungal and 1 bacterial genotypes, while the three transgenic lines were scored for a total of 11 fungal and 7 bacterial genotypes with 4, 4, and 3 specific fungal and 3, 4, and 0 specific bacterial genotypes for each line, respectively. Similarly, for transgenic and non-transgenic lines of AB-95, a total of 26 fungal and 15 bacterial genotypes were observed. The non-transgenic AB-95 line was scored for 8 fungal and 2 bacterial genotypes, while the three transgenic lines were scored for a total of 18 fungal and 13 bacterial genotypes out of which 5, 6, and 7 for fungi and 5, 4, and 4 for bacteria were scored, respectively.

**Table 4:** Specific microbial genotypes observed in fungal and bacterial RISA.

	**Variety**	**Line specific bands**				**Total**
Fungi	DN	DN (5)	DN-13 (4)	DN-120 (4)	DN-127 (3)	16
	AB-95	AB-95 (8)	AB-11 (5)	AB-18 (6)	AB-31 (7)	26
	Overall					42
Bacteria	DN	DN (1)	DN-13 (3)	DN-120 (4)	DN-127 (0)	8
	AB-95	AB-95 (2)	AB-11 (5)	AB-18 (4)	AB-31 (4)	15
	Overall					23

### Fungal and bacterial risa and cluster analysis

**Figure 6:**
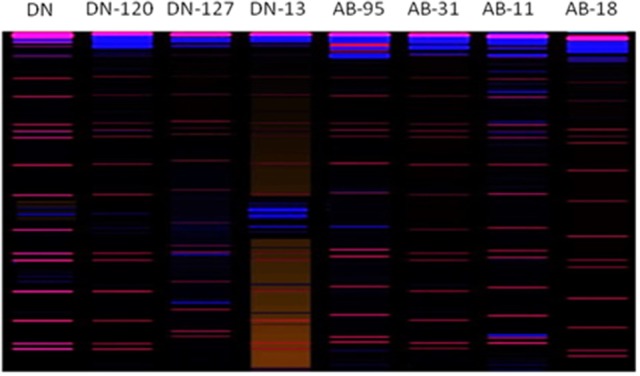
Digitized image (using 3130xl genetic analyzer with LIZ-500 internal size standard) of Fungal RISA. Red color bands indicate marker and blue color bands indicate RISA PCR product.

**Figure 7:**
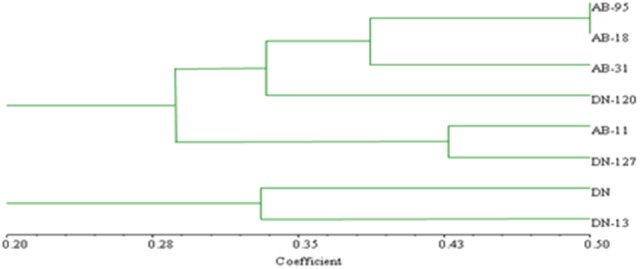
Clustering analysis using UPGMA method for fungal genotypic variation of RISA. AB-95 and DN are the parental non-transgenic *B. napus* varieties. AB-31, AB-11, AB-18, and DN-120, DN-127, DN-13 are their corresponding transgenic lines with *NiC* gene. The dice coefficient was estimated as similarity Index. The scale below dendrogram is of the similarity coefficients.

Bands scored during fungal RISA ranged from 100 to 600 bp on 6% denaturing polyacrylamide gel with LIZ-500 internal size standards (Figure 6). Binary data was obtained by scoring the banding pattern of fungal RISA and was used for computing in the Jaccard's similarity index. The similarity index values of all the fungal genotypes observed for each line (both non-transgenic and transgenic) were obtained by using computer software Ntsys 2.10. The similarity coefficient based on pair-wise comparison ranged from 0.20 to 0.50. The similarity index values were then used to construct dendrogram of fungal RISA products using the same software Ntsys 2.10 and their results are presented (Figure 7). A total of 2 major clusters were observed in the fungal RISA at a similarity coefficient difference of 0.3. In one cluster, there are total of 6 lines containing both transgenic and non-transgenic cultivars, while the second cluster contains only 2 lines of both transgenic and non-transgenic origin.

**Figure 8:**
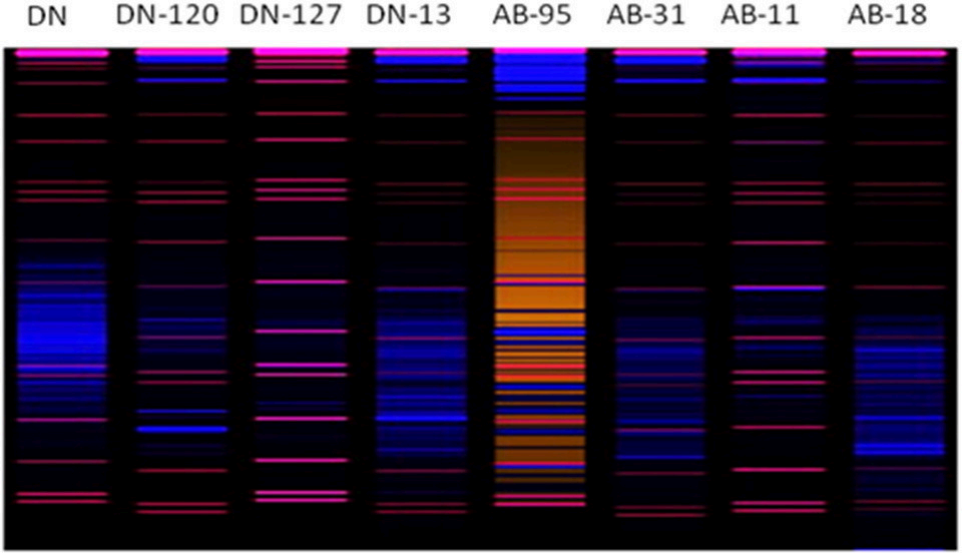
Digitized image (using 3130xl genetic analyzer with LIZ-500 internal size standard) of bacterial RISA. Red color bands indicate marker and blue color bands indicate RISA PCR product.

**Figure 9:**
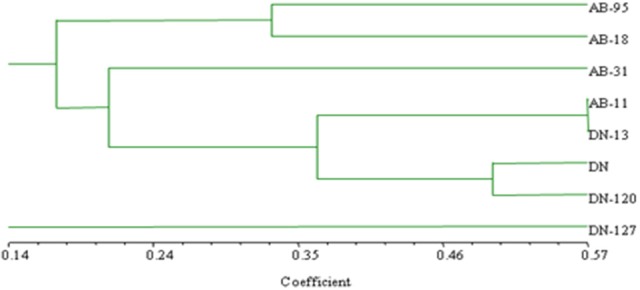
Clustering analysis using UPGMA method for bacterial genotypic variation of RISA. AB-95, and DN are the parental non-transgenic varieties. AB-31, AB-11, AB-18, and DN-120, DN-127 and DN-13 are their corresponding transgenic lines. The dice coefficient was estimated as similarity Index. The scale below dendrogram is of the similarity coefficients.

The bands, which were observed in the bacterial RISA of rhizosphere soil of both transgenic and non-transgenic lines of *B. napus* varieties, are shown (Figure 8). The similarity coefficient based on pair-wise comparison ranged from 0.14 to 0.57. The similarity index values were then used to construct dendrogram of bacterial RISA products using the same software Ntsys 2.10 and their results are presented (Figure 9). A total of 2 major clusters were observed in the bacterial RISA at a similarity coefficient difference of 0.00. In one cluster, there are total of 7 lines of both transgenic and non-transgenic varieties used in the experiment, while the second cluster contains only 1 line of DN.

The cluster analysis of both fungal and bacterial RISA indicated the grouping pattern of the fungal and bacterial genotypes which were found to be non-significantly impacted by the introduction of the transgene. No line specific or genotype specific clusters were observed between the transgenic and non-transgenic lines of both tested varieties. All the grouping patrons are equivocal and have greater substantial equivalence as compared to substantial variance.

### Analysis of molecular variance (AMOVA) of fungal and bacterial RISA

The binary data collected from bands of fungal and bacterial RISA were subjected to analysis of molecular variance (AMOVA) (Table 5). Source of variance were between the transgenic and non-transgenic lines and between the varieties. Genotypic structural differentiation was estimated based on genotypic variance with 100 permutations. F_GT_ represents the differentiation between transgenic and non-transgenic lines, while F_IT_ the differentiations between the varieties. The AMOVA results indicated no significant variation in the fungal as well-bacterial community structure between the rhizosphere of transgenic and non-transgenic lines. In the replicated experiments, the bacterial communities were recorded to be affected more by the transgene impact as compared to the fungal communities. The variation percentage of bacterial communities was recorded to be 12% while the fungal communities showed 4% variation. On the other hand, the bacterial community structures of DN and AB-95 varieties were severely impacted by the varietal differentiation which was recorded to be 79% while the fungal community structures were moderately impacted by the varietal differentiations. The variation percentage was recorded to be 54% for the fungal community structures of the two tested varieties.

**Table 5:** The analysis of molecular variance (AMOVA) for fungal and bacterial RISA.

	**Source of variance**	**Df**	**SS**	**MS**	**Comp. of variance**		**Genotypic structural differentiation**	***p*-value**
					**Est. Variance %**			
Fungal RISA	Between transgenic and non-transgenic	3	3.78	0.94	0.13	4%	F_GT_ = 0.00	0.74
	Among the varieties	1	13.78	13.78	1.42	54%	F_IT_ = 0.56	0.01
	Total	4	17.56	14.72				
Bacterial RISA	Between transgenic and non-transgenic	3	5.11	1.28	0.22	12%	F_GT_ = 0.27	0.13
	Among the varieties	1	54.06	54.06	5.86	79%	F_IT_ = 0.88	0.01
	Total	4	59.17	55.34	6.08			

## Discussion

### Impact of the transgene on fungal resistance

The unintended impact of the synthetic chitinase (*NiC*) gene was evaluated on the rhizosphere microbial diversity and enzymatic activities between the *B. napus* non-transgenic DN and AB-95 varieties and their corresponding transgenic lines. The synthetic chitinase (*NiC*) gene in transgenic lines is driven by the constitutive *CaMV35S* promoter (Khan et al., 2013). The transgene encodes chitinase enzyme that digests the chitin protein present in the cell wall of different microbes and cause calamitous impact on their growth. The introduction of such a gene and the encoded protein in the plant system may impact many biotic and abiotic factors including plant invasiveness potential, gene transfer ability, and rhizosphere microbial community structure and functions. Changes in the rhizosphere microbial communities may in turn, alter the soil biochemical structure through interactions with root exudations, tiller degradations, nutrient cycling and bioremediations (Lemanceau et al., 1995; Siciliano et al., 2001; Graner et al., 2003; Garbeva et al., 2004; Jousset et al., 2006; Rasche et al., 2006). Therefore, impact of the transgene in the transgenic lines of both *B. napus* varieties was evaluated for substantial equivalence with the non-transgenic control plants for antifungal assay, rhizosphere microbial community structure and their enzymatic activities.

The disease causing fungus, *A. brassicicola* was confirmed through microscopic observation and PCR analysis. The antifungal assay was performed to observe whether the *NiC*-transgenic lines confer resistance to the fungal pathogen. Our results indicated no antifungal activity in the non-transgenic lines, whereas all the transgenic lines showed different levels of antifungal activities as growth inhibition of *A. brassicicola*. Two lines, DN-120 and AB-31 showed significantly higher fungal growth inhibition. This might be due to the high expression of the *NiC* gene in these two lines as compared to other transgenic lines. Similar results of antifungal activity were observed in *NiC*-transgenic plants against *Fusarium oxysporum* (Kong et al., 2014). Transgenic potato plants, transformed with chitinase (*ChiC*) gene also exhibited resistance against fungal pathogens, *Botyrytis cinerea* (gray mold), *Alternaria solani* and *F. oxysporum* (Khan et al., 2008, 2011).

### Rhizosphere enzyme activity assay

It is generally agreed that genetic changes in plants either through conventional breeding or transgenic technology may affect rhizosphere microorganisms through root exudations and changes in the soil biochemical processes (Siciliano and Germida, 1999; Saxena and Stotzky, 2001; Schmalenberger and Tebbe, 2003). The results of the present study indicated non-significant variation between the soil enzymes activities of the transgenic lines compared with their non-transgenic competitors. However, inconsistent results were observed in terms of variations in one of the replicated trails of the study. Some soil enzyme activities were found to be relatively varied from their non-transgenic parental lines such as alkaline-phospho-monoestarase activity was found to be relatively higher in AB-18 and AB-31 lines. Arylsulphatase, β-glucosidase, urease and sucrase activities were found to be substantially equivalent. Moreover, we did not find any significant or clear impact of the variable gene expression levels on the enzyme activities between transgenic lines and their wild type control plants of both DN and AB-95 varieties. Our results were in complete agreement with those of Flores et al. (2005). They reported that the soil enzymes activities were not consistently different in soil mixed with biomass of Bt or non-Bt corn. Non-significant differences were found in the activities of several enzymes between soils of Bt and non-Bt cotton plants (Shen et al., 2006). On the contrary, the activities of dehydrogenases, methanogenesis and phosphatases increased in flooded soil with addition of straw of transgenic Bt-rice (Wu et al., 2004a,b). Similarly, the activities of acid phosphomonoesterase, cellulase, invertase and soil urease were increased and that of arysulfatase was decreased after addition of plant parts of two Bt cotton varieties to the soil (Sun et al., 2007). Along the transgene impact, such effects may also be caused by different biotic and abiotic stimulants including varied root exudates, altered plant watering timings, and water and nutrients uptake in different fashions. Interpreting such minor changes is difficult because minor variations in the biochemical composition could have a vivid impact on the soil environment (Mimura et al., 2008), but occurrence of such minute line-specific biochemical activities in soil is not enough to assess statistical significance in these types of analysis. Results of the present study were found similar to activities of acid phosphatases in soil with transgenic papaya (Wei et al., 2006) that differed from the rest of soil enzymes. This may be attributed to soil physico-chemical properties, soil enzyme origin and plant type (Edwards, 2002; Andersen, 2003).

### Microbial community structures

No significant variations were observed in the rhizosphere fungal and bacterial community structures of both transgenic and non-transgenic lines. Several studies have found that the diversity of soil enzyme activities and microbial communities may be affected by plants. However, the results of these studies were not consistent regarding impact of transgenic plants on the soil enzyme activities, rhizospheric soil nutrient cycling, and indigenous microbial populations (Saxena and Stotzky, 2001; Castaldini et al., 2005; Chen et al., 2012; Wei et al., 2012). Risk assessment studies on the impact of *Bt* genes on rhizosphere microbial communities are reviewed (Turrini et al., 2015). For example, the Cry1Ab protein production in transgenic corn and its release to soil from root exudates or biomass had no significant effect on culturable bacteria, fungi, and protozoa (Saxena and Stotzky, 2001; Flores et al., 2005). These studies may be used as helping material for environmental assessment of chitinase genes as both are isolated from soil bacteria. A few studies also reported the effect of chitinases on rhizosphere glomeromycota in transgenic rice and *Nicotiana sylvestris* plants. Transgenic rice with chitinase production showed reduction in root colonization by endophytic and mycorrhizal fungi, whereas exhibited an increase in intraradical bacteria (Yang et al., 2002). On the contrary, the micorrhizal colonization was not affected in the roots of transgenic *N. sylvestris* with chitinase production (Vierheilig et al., 1993). In the present study, similar results were found which indicated non-significant impact of the transgene and its relative expression levels on the corresponding biochemical and microbial structure of the rhizosphere. However, some genotypes of fungi as well as bacteria were found to be specific to the transgenic lines and some genotypes were only present in the non-transgenic lines, though only in one or two of the replicated experiments.

### Impact of plant genotype

Another objective of the study was to assess the variance of soil enzyme activities and soil microbial diversity between the two varieties of *B. napus* (DN and AB-95). It is predominantly accepted that plants and soil microbes have co-evolved in association for long time and are highly important for each other. Plants may have effects on the rhizosphere microbial populations as a result of chemicals exchange in the shape of root exudates (Bais et al., 2006; Haichar et al., 2008). Microbial populations and composition of root exudates varies from plant to plant in the vicinity of the root (Somers et al., 2004). There is no confusion in the understanding that factors such as soil properties and plant species greatly influence the composition, structure and function of corresponding microbial communities. But the extent to which these factors influence and contribute to harbor microbial communities is not yet fully understood. Studies of cultivation-dependent methods provided the first insight of different composition of the bacterial community on and beside plants (Kremer et al., 1990; Lemanceau et al., 1995; Maloney et al., 1997). In one study, even cultivar dependent relationship in plant–microbes interactions was revealed (Germida and Siciliano, 2001). The results of the present study also indicated that the type of plant genotype/variety strongly influences the microbial community structure as well as the enzyme activities in the soil of the two varieties of *B. napus*. Bacterial community structures of DN and AB-95 varieties were severely impacted by the varietal differentiation, which was recorded as 79%, whereas, the fungal community structures were moderately impacted by the varietal differentiations. The variation percentage was recorded to be 54% for fungal community structures of the two tested varieties. Similar results of altered microbial structures were found for different old and modern wheat cultivars (Graner et al., 2003). The old wheat cultivars were reported to be colonized by phylogenetically diverse rhizobacteria as compared to the rhizosphere of modern cultivars, dominated by swift Proteobacteria. Cultivar specificity was also reported for oilseed rape (Rengel et al., 1998). Similar results of difference and variance were found for composition of the root-associated microbial community of transgenic and non-transgenic canola cultivars (Siciliano et al., 1998; Siciliano and Germida, 1999). Several studies on transgenic corn harboring the Cry1Ab protein showed variations in the microbial and microfaunal communities at the field and greenhouse levels (Griffiths et al., 2005, 2006). These variations were attributed to different non-Bt corn cultivars, different crops, soil type, and stage of plant growth.

Along the rhizospheric microbial community structures of *B. napus* varieties, soil enzyme activities were also significantly affected by the varietal differences. Except for β-glucosidase, urease and sucrase, results of arylsulphatase and phosphomonoestarase of DN variety were observed to be highly variant from those of the AB-95. The probability values according to the *t*-test between their variance were recorded to be 0.09 for alkaline phosphomonoestarase and 0.05 for arylsulphatase, reflecting substantial variance. The soil enzymes results were found to be highly inconsistent and because of soil enzymes importance for the growth of microorganisms, these act as a good indicator of assessing the overall microbial activity (Dick, 1994). Many reports have showed that soil enzyme activities interacted positively with the diversity and productivity of plant communities, and increased with vegetation development (Hattori et al., 1997; Waldrop et al., 2000; Mamilov et al., 2001; Smith et al., 2002).

Crops are grown in agricultural fields, where these are exposed to several biotic and abiotic environmental fluctuations, which in turn may cause varied experimental outcomes during environmental risk assessment. Risk assessment studies conducted on herbicide resistant transgenic plants were reported with lesser or even no unintended effects on the surrounding biota (Schmalenberger and Tebbe, 2002, 2003). However, some studies on *B. napus* with qualitative gene products were found to have significant impact on soil enzymes and soil microbial diversity (Siciliano et al., 1998; Siciliano and Germida, 1999; Dunfield and Germida, 2001). These results were found to be inconsistent and were regarded as temporary. On the contrary, some studies revealed consistent significant variations in soil enzyme and physiochemical properties between transgenic and non-transgenic plants but these differences were attributed to different seasons and crop varieties rather than the transgene impact (Zeng et al., 2016). Thus, owing to the biological and ecological variation in the fields, long term and continuous cultivations data must be obtained for further evaluation of the transgene impact and proper management of the transgenic crops (National Research Council, 2002).

## Conclusion

In conclusion, results of the present study indicated non-significant impact of the *NiC* transgenic *B. napus* on the rhizosphere microbial diversity and soil enzyme activities. However, these results are obtained under controlled growth environment of the greenhouse. The long term impact of the transgene on the tested parameters must be further assessed under more realistic environmental conditions such as special netted-house and confined filed.

## Author contributions

MK and SS equally contributed to this manuscript. AJ and IM provided technical help throughout the research and checked the manuscript critically for grammatical errors.

### Conflict of interest statement

The authors declare that the research was conducted in the absence of any commercial or financial relationships that could be construed as a potential conflict of interest.
